# Case Report: Metaplastic breast carcinoma with osseous and chondrodifferentiation in liver metastasis: a rare case and review of literature

**DOI:** 10.3389/fonc.2025.1437887

**Published:** 2025-10-02

**Authors:** Wenfang Li, Qin Ou, Tian-xiang Zhang, Bin Bin Long, Ke Zhou, Lun Hua Zhao

**Affiliations:** ^1^ Department of General Surgery, the Taihe Hospital Affiliated to Hubei University of Medicine, Shiyan, Hubei, China; ^2^ Department of Pathology, Hubei University of Medicine, Shiyan, Hubei, China

**Keywords:** breast oncology, metaplastic carcinoma, neoadjuvant chemotherapy, mastectomy, thoracic oncology

## Abstract

**Background:**

Metaplastic carcinoma of the breast with mesenchymal differentiation (MCMD) is a type of metaplastic breast carcinoma (MpBC) that is very rare and aggressive. The present case provides valuable information for clinicians on this MpBC.

**Case presentation:**

A 41-year-old woman visited our hospital for a palpable painless mass in the left breast. Core needle biopsy (CNB) was performed, and the pathological result was infiltrating ductal carcinoma. Epirubicin (100 mg/m^2^) + cyclophosphamide (600 mg/m^2^) for four cycles was given. Color Doppler ultrasound examination indicated no obvious change in the size of the left breast mass. We changed to paclitaxel (175 mg/m^2^) for two cycles. Re-examination on April 26, 2018 with color Doppler ultrasound indicated that the tumor diameter increased to 8.39 cm × 8.07 cm × 6.19 cm. Radical resection of the left breast carcinoma was performed on June 04, 2018. The postoperative pathological results showed that the left breast tumor was composed of carcinoma and sarcoma components, without nerves and vascular invasion. The immunohistochemistry results were as follows: ER: (−), PR: (−), HER2: (−), CK5/6 (+), CK7: (+), E-cadherin (+), Ki67: 40% (+), P120: (+), P53 diffuse +, P63: (+), and S100 partially positive, GATA-3: (+). Four cycles of vinorelbine (25 mg/m^2^) + cisplatin (40 mg/m^2^) were performed after the operation. Enhanced CT indicated a 6.0 cm × 4.6 cm mass in the liver on January 1, 2019 through regular review, and liver lobectomy confirmed that metastasis originated from sarcoma components, together with bone and cartilage differentiation. The immunohistochemistry results indicated the following: ER (−), PR (−), GATA-3 (−), CD34 (+), P63 (−), CK8 (−), P40: (−), and vimentin: (+). The patient received oral anlotinib 12 mg once a day, with 2 weeks on/1 week off for eight cycles. The patient survived and showed no signs of recurrence at the follow-up visit.

**Conclusion:**

This case indicated that CNB may not always give an accurate diagnosis for MCMD. Neoadjuvant chemotherapy with epirubicin, cyclophosphamide, or paclitaxel for MCMD may not be effective for patients showing no sensitivity to these drugs. In addition, regular postoperative follow-up plays an important role in the early detection of remote metastasis, and timely surgical excision of a single metastatic lesion in the liver can lead to long-term progression-free survival (PFS).

## Introduction

Metaplastic breast carcinoma (MpBC) is a rare subtype of breast cancer, defined as mammary carcinoma with squamous or mesenchymal differentiation, that may include spindle cell, chondroid, osseous, or rhabdomyoid differentiation patterns (1), accounting for 0.2%–1% of breast carcinomas (2). MpBC patients had worse prognosis than those with triple-negative breast cancer (TNBC). In a multivariate analysis, MpBC had approximately twice the risk of local recurrence than TNBC (3). However, it was reported that MpBCs correlated with improved progression-free survival (PFS) and overall survival (OS) compared to TNBC (4). Data about treatment options and outcomes are still controversial for this rare disease. MpBCs could be histologically classified into six distinct groups based on the World Health Organization 2019 classification of breast tumors: (i) low-grade adenosquamous carcinoma, (ii) fibromatosis-like metaplastic carcinoma, (iii) spindle cell carcinoma, (iv) squamous cell carcinoma, (v) metaplastic carcinoma of the breast with mesenchymal differentiation (MCMD), which included chondroid, osseous, and other types of mesenchymal differentiation, and (vi) mixed metaplastic carcinoma (5).

Due to a rare subtype of breast cancer that affects only a small proportion of breast cancer patients, no clinical trials exist in particular. Therefore, treatment options for MpBC require further accumulation of experience (6). Due to its rarity, evidence for MpBC is primarily derived from case reports, case series, and retrospective analysis. MCMD displays with osseous/chondroid differentiation and is rare with great challenges in diagnosis and treatment (7). Here we report a 41-year-old female patient diagnosed with MCMD and try to supply valuable information for this rare disease.

## Clinical data

**Figure 1:**
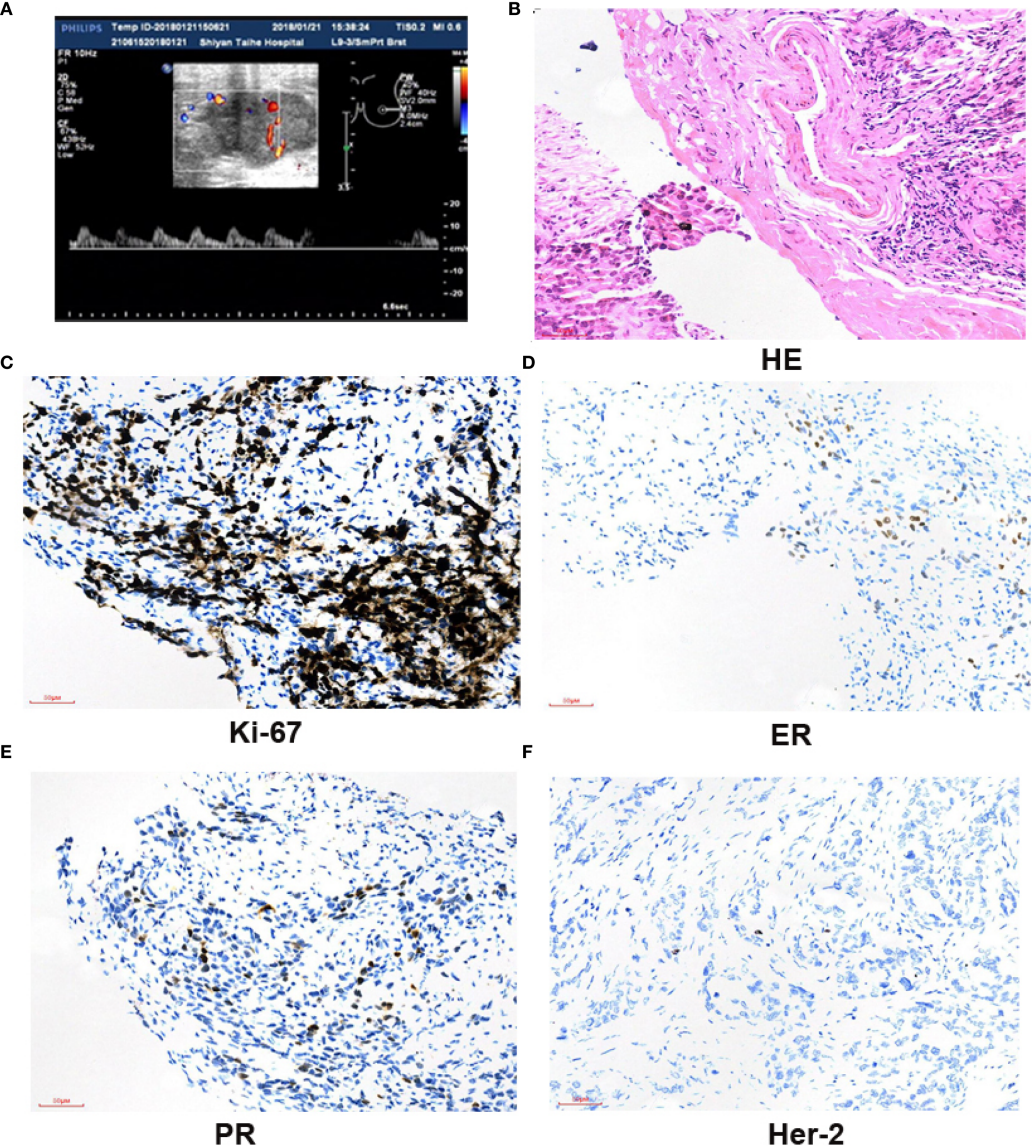
A breast mass in the left breast was biopsied; immunohistochemistry results. **(A)** Breast US images revealed a hypoechoic irregular mass in the left breast, with a size of about 40 × 35 × 24 mm. The boundary was uneven, and no strong punctate echo was observed. **(B)** Core needle biopsy of the mass (H&E, ×200); tumor cells displayed with a cord-like pattern, with some in a nest-like arrangement. The tumor cell nuclei are large, and scattered mitotic images could been observed. Dense fibrous connective tissue in the tumor stroma, eosinophilic staining, and lymphocytes could be observed around. Scale bar = 50 µm. **(C)** The positive staining of Ki-67 in cells is about 50% (×200). Scale bar = 50 µm. ER30%, moderate; PR10%, moderate; HER2 was negative (×200). Scale bar = 50 µm **(D–F)**.

**Figure 2:**
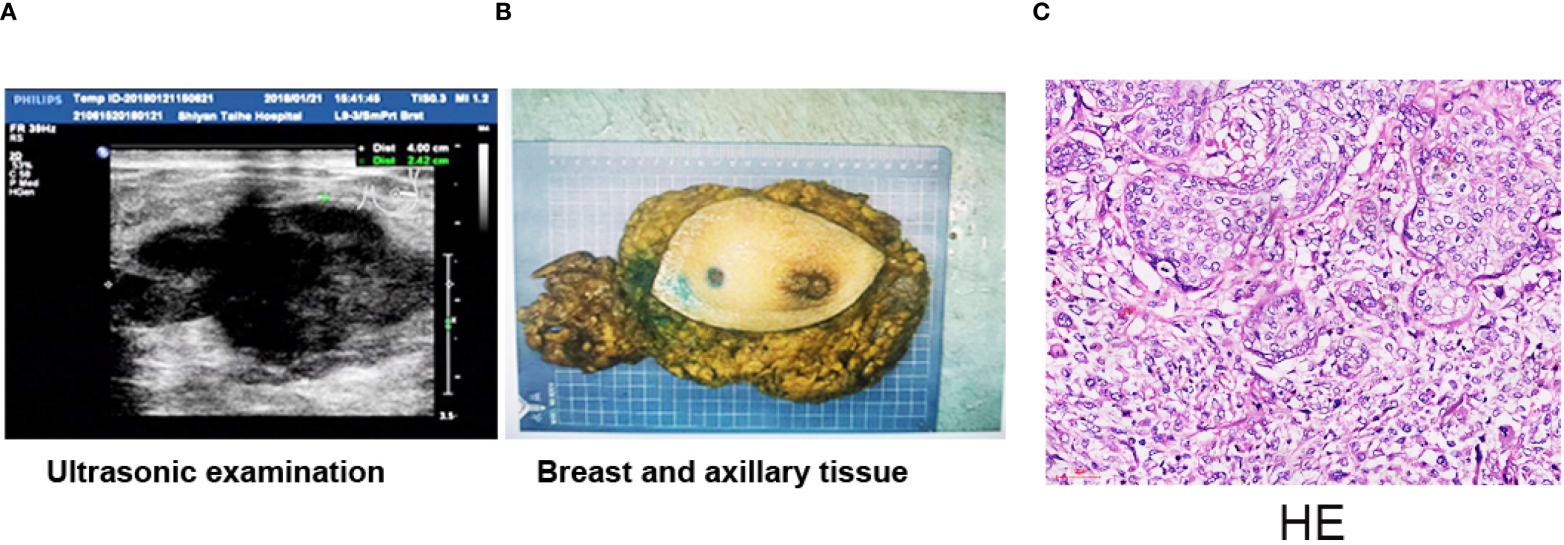
Breast mastectomy and H&E staining result of the tumor. **(A)** Breast US images revealed a hypoechoic irregular mass in the breast left upper quadrant, with a size of about 8.39 × 8.07 × 6.19 cm. **(B)** Whole excised tissue of the left breast. The mass was completely moved out with breast mastectomy. **(C)** The tumor was composed of two components: carcinoma and sarcoma components. The tumor cells in the cancer area are epithelial-like, locally nested, nuclear vacuolated, and scattered mitotic images. The tumor cells in the sarcoma area are scattered, of varying sizes, round or oval, and with large, vacuolar nuclei. Multinucleated tumor giant cells are often observed, and mitotic images are more common showing moderate to marked nuclear pleomorphism. Scale bar = 50 µm (H&E, ×200).

The Ethics Committee of Taihe Hospital Affiliated to Hubei University of Medicine approved the protocol, and written informed consent was provided by the patient involved. The patient, ××× (represent the patient name), a 41-year-old woman, presented with “left breast mass more than 1 month ago.” On January 20, 2018, she visited the Breast Surgery Department of Taihe Hospital, Hubei University of Medicine. In the past 1 month, she felt that the lump was larger than before without any pain or nipple discharge. The patient denied a history of hypertension and any drug allergies. Upon clinical physical examination: a hard mass approximately 4.0×3.0 cm could be detected in the left lower outer quadrant 4 cm away from the nipple, without obvious tenderness. The surface was uneven, the boundary was unclear, and the range of motion was limited. No abnormality exists in the contralateral breast, and no obvious mass in the bilateral axilla was observed. The ultrasound examination on January 22, 2018 revealed a hypoechoic mass measuring 40 mm × 35 mm × 24 mm in the left outer lower quadrant of the breast, classified as BIRADS (breast imaging reporting and data system) VIa (**Figure 1A**). Core needle biopsy (CNB) was performed at our hospital, and the pathological report indicated infiltrating ductal carcinoma (IDC), with tumor cells exhibiting mild morphology and mild atypia (**Figure 1B**). The immunohistochemistry results showed ER positive at 30%, moderate; PR positive at 10%, moderate; and Ki-67 (labeling index: approximately 50%), and HER-2 was negative (**Figure 1C–F**). No distant metastases were found in the brain, liver, or lung upon computed tomography (CT) examination. Preoperative neoadjuvant chemotherapy was administered, consisting of four cycles of epirubicin (100 mg/m^2^) + cyclophosphamide (600 mg/m^2^). The color Doppler ultrasound examination indicated no obvious change about the size in the left breast mass. Then, we changed to paclitaxel (175 mg/m^2^) for two cycles. At re-examination on April 26, 2018 with color Doppler ultrasound, the tumor diameter in the left breast increased to 8.39 × 8.07 × 6.19 cm, with irregular shape, punctiform strong echo, and axillary lymph nodes at 12 × 7 mm (**Figure 2A**). Surgery was performed as decided.

Radical resection of the left breast carcinoma was performed on June 4, 2018. The postoperative pathological examination indicated that the left breast tissue was completely removed (**Figure 2B**). The size of the mass was 8.5 cm × 8.0 cm × 6.2 cm, and the section was gray and slightly hard. The tumor was composed of two components—carcinoma and sarcoma components—displayed with obvious mitotic images and nuclear polymorphism of unusual cellular or stromal components (**Figure 2C**). The immunohistochemistry results were as follows: ER: (-), PR: (-), HER-2: (-), CK5/6 (+), CK7: (+), E-cadherin (+), Ki67: (Labeling index: about 40%), P120: (+), P53 diffuse +, P63: (+), S100 partially positive, and GATA-3: (+). Four cycles of vinorelbine (25 mg/m^2^) + cisplatin (40 mg/m^2^) regime were performed after operation.

**Figure 3:**
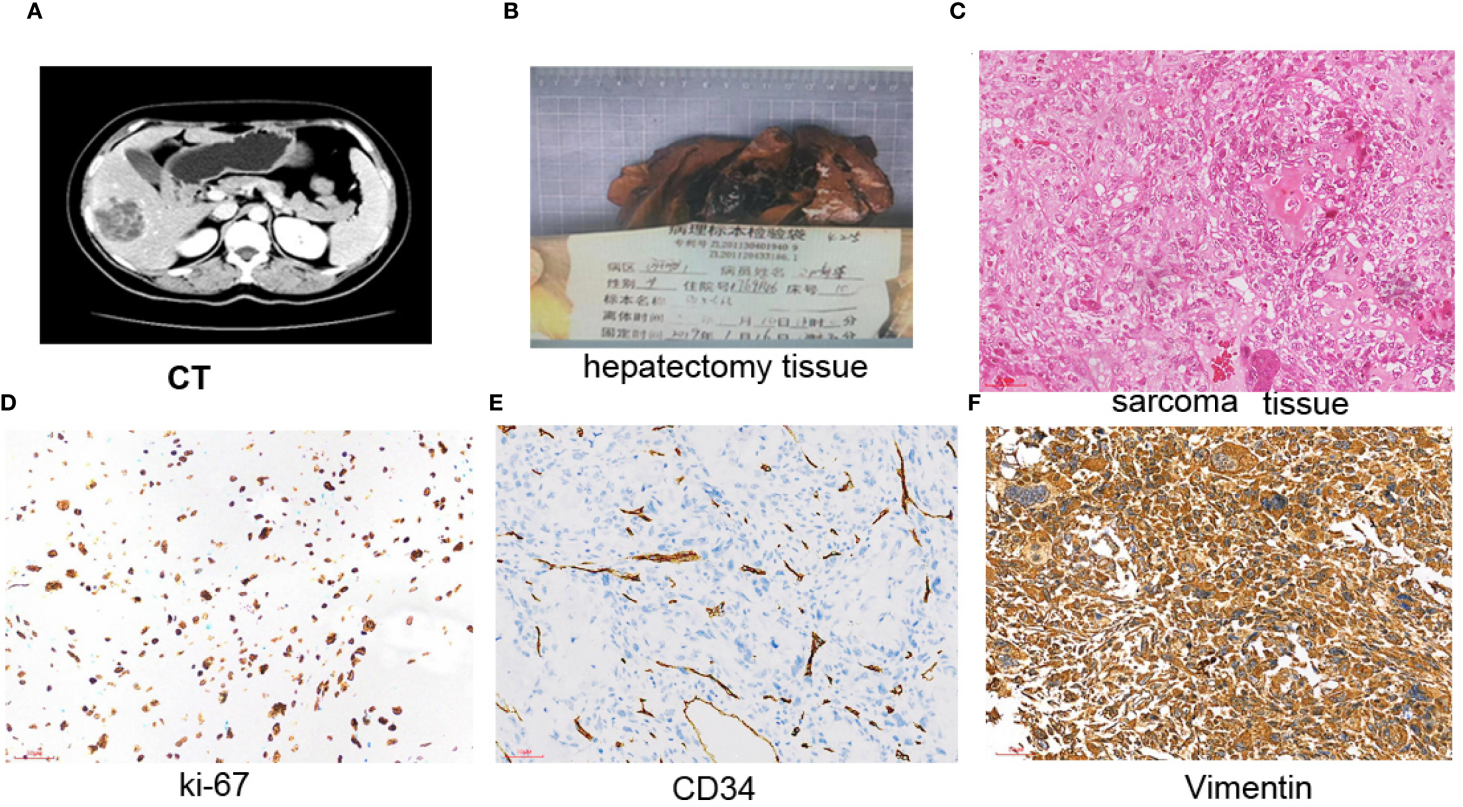
Liver metastasis was excised; immunohistochemistry results. **(A)** Enhanced CT indicated a 6.0 × 4.6-cm mass in the right lobe of the liver. **(B)** Whole excised hepatectomy tissue. **(C)** The tumor was composed of sarcoma components. The tumor cells are scattered, of varying sizes, round or oval, and with large and vacuolated nuclei. Displayed with obvious mitotic images and nuclear polymorphism. Multinucleated tumor giant cells are often observed, and mitotic images are common, with one to four small nucleoli. H&E, ×200. Scale bar = 50 µm. **(D)** Ki67 positive staining was about 50% in the nucleus in sarcoma components (×200). **(E)** CD34 was positive in stromal tissue (×200). Scale bar = 50 µm. **(F)** Positive staining with vimentin in the membrane and nucleus was observed in sarcoma components (×200). Scale bar = 50 µm.

Enhanced CT revealed a 6.0 × 4.6-cm mass in the right lobe of the liver on January 1, 2019 during the follow-up day (**Figure 3A**). Liver lobectomy was performed and confirmed the metastasis (**Figure 3B**). The H&E examination demonstrated that the metastasis was originated from sarcoma components (**Figure 3C**). The tumor formed with bone and cartilage differentiation, and bone trabeculae have been observed without nerves and vascular invasion. The immunohistochemical results indicated the following: ER (-), PR (-), GATA-3 (-), CD34 (+), P63 (-), CK8 (-), P40: (-), vimentin: (+), and Ki-67 (labeling index: about 50%), which confirmed that the metastasis originated from sarcoma components (**Figures 3D–F**). The patient received oral anlotinib 12 mg once a day, with 2 weeks on/1 week off for 8 cycles. The patient still survived without any new sign of recurrence until the follow-up day.

## Discussion

### Classification of MpBC

MpBC is a group of heterogeneous malignancies characterized by non-glandular differentiation of the breast tissue, including osseous, sarcomatous, or squamous histology (8). The presence of distinct antigens, such as cytokeratins (34βE12, CK5/6, CK14, and CK17), luminal cytokeratins (CK8/18, CK7, and CK19), and vimentin (mesenchymal cells) or myoepithelial cell markers (S-100 protein, actin, and high-molecular-weight cytokeratin) could establish the differentiated diagnosis of MpBC (9). MpBCs are classified as monophasic (only one metaplastic component) or biphasic (both metaplastic and non-metaplastic components) tumors. The former includes pure spindle cell carcinoma (SpCC) and squamous cell carcinoma (SqCC), and the latter includes mixed MpBC and MCMD (10).

However, the classification of different subtypes of MpBC could be quite challenging. Several studies suggest that SqCC is the most commonly encountered subtype of MpBC (11). MCMD is a type of MpBC displayed with osseous and/or chondroid differentiation. It is demonstrated that the mesenchymal subtype was significantly associated with worse 5-year disease-free survival and disease-specific survival (12).

### Diagnosis of MpBC

It is well established that CNB is the gold standard for the differential diagnosis of breast lesions, with both high sensitivity and specificity (13). However, it poses significant diagnostic challenges for MpBC by CNB, as the pathological diagnosis of MCMD requires the demonstration of epithelial and heterologous (chondroid or osseous) (14). Usually, MCMD does not express ER, PR, or HER2; thus, it can often be evaluated as a subgroup of TNBC (15). ER, PR, or HER2 could also expressed in MpBC. This is in line with our CNB immunohistochemistry results which appeared with 30% ER expression (16). However, the postoperative pathological examination and immunohistochemistry results of the left breast tumor showed ER: (-), PR: (-), and HER-2: (-). The hormone receptors in the CNB results were different from the postoperative pathological examination. This should relate to tumor heterogeneity.

In our case, the initial diagnosis was IDC by CNB, the reason might be that CNB had not gained enough tissues that contained stromal components, so a ductal or lobular adenocarcinoma was diagnosed. Our results indicated the diagnostic limitations of biopsy in rare histological subtypes and reminded clinicians to remain vigilant for the potential histological heterogeneity. Even this is very infrequent; the clinician should be aware of this complexity.

### Neoadjuvant and systemic chemotherapy of MpBC

The role of neoadjuvant chemotherapy (NAC) in this setting remains unclear as MpBCs are relatively chemotherapy-refractory. Henessy et al. reported a low rate of 10% for pathological complete response (pCR) in patients with MpBCs following neoadjuvant chemotherapy (17). Han et al. reported 17% patients who achieved pCR in MpBCs received neoadjuvant chemotherapy (18). However, another study indicated that 23% of pCR rate was observed in MpBC patients and suggested that NAC should be considered (19). Systemic therapy and/or radiotherapy (RT) have been considered as components of the definitive treatment in most MpBC patients due to the negative prognosis associated with the histology and the increased likelihood of advanced-stage disease at presentation (2).

Studies have demonstrated variable responses to systemic therapy. It is reported that some MpBC cases responded poorly to systemic chemotherapy and had a poor prognosis, while some demonstrated better outcomes with chemotherapy (20, 21). As far as systemic therapy is concerned, several studies emphasized that the response of an appropriate chemotherapy regime depends on the histological type of MpBCs (22–24). As shown in **Table 1**, Chen et al. reported a modest response to taxane-based therapy (25). Moreover, cases with a squamous epithelial component showed a good response to paclitaxel and cisplatin-based chemotherapy regimens, while cases with sarcomatous elements responded to doxorubicin and ifosfamide-based regimens (26–30). In our patient, the tumor diameter suddenly increased even if doxorubicin, ifosfamide, or paclitaxel chemotherapy was given. This demonstrated that it was chemotherapy-refractory to these drugs. Our case report demonstrated that MCMD might be completely chemotherapy-refractory, which is inconsistent with the sensitive results mentioned earlier. This supplied valuable treatment experience for chemotherapy for this disease.

**Table 1:** Chemotherapy regime responses on the histological type of MpBC.

Author	Subtype of MpBC	Age	Chemotherapy drug	Sensitivity
Chen et al., 2011 (25)	SCC	48	Vinorelbine + cisplatin	Resistant
	SCC	47	Epirubicin + cyclophosphamide	Resistant
Haruko Takuwa et al., 2014 (26)	MCMD	59	Doxorubicin + cisplatin + cyclophosphamide	Near pCR
Pandey A et al., 2019 (23)	SCC	39	Doxorubicin + cyclophosphamide + taxol	pCR
Alan O et al., 2019 (27)	SCC	72	Weekly paclitaxel+ epirubicin + cyclophosphamide	pCR
Vranic S et al., 2020 (28)	SpCC	42	Cisplatin	Resistant
Noro A et al., 2022 (29)	SCC	40	Paclitaxel	pCR
Tower A et al., 2023 (22)	Mixed MBC	68	Adriamycin + cyclophosphamide + taxol	Near pCR
Huang C et al., 2023 (24)	MCMD		Docetaxe + epirubicin + cyclophosphamide	Resistant
Fouad Nahhat et al., 2024 (30)	SpCC	40	Adriamycin + cyclophosphamide	pCR

SCC, squamous cell carcinoma; MCMD, metaplastic carcinoma of the breast with mesenchymal differentiation; SpCC, spindle cell carcinoma; mixed MBC, mixed metaplastic carcinoma; pCR, pathological complete response.

### MpBC liver metastasis

MpBC shows a tendency for early hematogenous spread to distant organs such as the lung, liver, and bone, while local recurrence is also quite frequent (31). On the contrary, the rates of axillary metastasis might vary depending on tumor morphology. The hematogenous spreading route is particularly more common in subtypes with predominant sarcomatoid carcinoma in the spectrum. Acar et al. reported that the risk of distant metastasis was higher in MpBC patients than in patients with ductal or lobular adenocarcinomas, while the risk of lymph node involvement was lower in MpBC patients (32). Yamaguchi et al. studied the prognosis of 53 cases of distinct subtypes of MpBC and showed that patients with high-grade spindle carcinoma or squamous carcinoma were at a higher risk of recurrence and developing distant metastasis compared to patients with other MpBC subgroups (33). Only four cases of MpBC with pulmonary metastasis have been reported (34). It has been reported that approximately 50% of MpBC patients develop distant metastasis after the primary surgery, with lung and brain being the organs most commonly involved (35).

In our case, the patient developed liver metastasis, while axillary lymph node was not involved. The pathological examination results confirmed that the metastasis was of sarcoma components. Our results revealed that MCMD had the potential to undergo hematogenous metastasis to the liver. When cancer cells from the primary tumor acquired mesenchymal characteristics, they exhibit enhanced migratory capabilities and increased resistance to conventional therapies (36).

Clinicians and researchers must remain vigilant about the potential for hematogenous spread in patients diagnosed with MCMD. In our patient, the metastatic lesion in the liver was excised successfully, and there was no other metastasis lesion identified until the follow-up day. These suggest that regular monitoring could aid in detecting liver metastasis at an early stage, and timely operation should be adopted when a single metastatic lesion happened in the liver.

### Mastectomy on MpBC

MpBC patients usually present with a large breast mass, which indicates a locally advanced disease, and typically patients are not candidates for breast-conserving surgery. The prognosis of MpBC is even poorer compared to TNBC. A study comparing the outcomes of 5,142 MpBC and 50,705 TNBC patients suggested that the 5-year overall survival rate of MpBC was 55%, which was less than that of TNBC with an overall survival rate of 72% (37). Therefore, modified radical mastectomy or mastectomy with or without axillary dissection could be implemented (27).

In our patient, the tumor diameter increased to 8 cm suddenly, modified radical mastectomy was adopted, and no recurrence was observed in the breast or axilla in the later time. These suggest that mastectomy was necessarily carried out promptly even if the tumor diameter was large enough.

### Molecular mechanisms on MpBC

It was observed that distinct transcriptomic alterations such as hypoxia-related and immune-related genes contributed to the pathogenesis in MpBCs (36). Significant heterogeneity in the expression of PD-L1 in spindle and squamous cell carcinoma subtypes might be a more promising immunotherapy target (38). A case of a 71-year-old female patient with high-grade MpBC successfully achieved a complete pathologic response using pembrolizumab, paclitaxel, carboplatin, adriamycin, and cyclophosphamide in the manner previously reported by the Phase III Keynote-522 clinical trial (39). The effect of ipilimumab and nivolumab that dually targeted CTLA-4 and PD-1 was associated with exceptional responses in a subset of patients versus no activity. This combination warrants further investigation in MpBC, with special attention to understanding the mechanisms (40).

The epithelial–mesenchymal transition (EMT) had participated in the malignant progression of MpBC. The aberrant expression of Snail, a transcription factor that downregulates epithelial genes and is mostly observed in metaplastic carcinomas with chondroid difference, leads to changes in epithelial architecture and induction of EMT and increases the risk of carcinogenesis and metastasis (41). In our patient, a high level of vimentin was observed in liver metastasis lesions, and this demonstrated that the sarcoma components in MCMD acquired metastasis ability through EMT.

Several mutations have been identified in MpBC—for example, p53 plays significant roles in cell cycle disruption and carcinogenesis, leading to a more aggressive phenotype and drug resistance in MpBC (38). Somatic mutations in genes such as p53, phosphatidylinositol-4, 5-bisphosphate 3-kinase catalytic subunit alpha (PIK3CA), breast cancer gene (BRCA), DNA topoisomerase II alpha (TOP2A), or more rarely phosphatase and tensin homolog (PTEN) have also been related to MpBC development (42). Furthermore, activation of the Wnt/β-catenin signaling pathway, which has been associated with dysregulated immune responses and cell cycle disruption, has been observed in MpBC cases (43). In our case presentation, high levels of p53 and vimentin have been observed. This suggested the high malignancy of MpBC. These molecular findings suggested not only a novel pathway of carcinogenesis but also potential therapeutic targets specific to each MpBC pathological subtype.

## Conclusion

In summary, MpBC presents very rare, unique features which differentiate it from ductal carcinoma of the breast. The diagnosis should be confirmed by immunohistochemistry, which also plays a crucial role in the classification of subtypes. Even if she had not responded to neoadjuvant or systemic chemotherapy, our patient successively acquired primary breast surgery and liver metastasis lesion excision, with no recurrence happening until the follow-up day, which implied important clinical information for this disease. However, this is the therapeutic experience of only one patient. This could not reflect the whole properties of this disease. Further studies are necessary to fully comprehend the complexities of this disease and to devise effective interventions. Better understanding of the molecular pathways involved in cancer development would contribute to the development of individualized therapy.

## Data Availability

The datasets presented in this study can be found in online repositories. The names of the repository/repositories and accession number(s) can be found in the article/supplementary material.

## References

[1] Narayan P Kostrzewa CE Zhang Z O’Brien DAR Mueller BA Cuaron JJ. Metaplastic carcinoma of the breast: matched cohort analysis of recurrence and survival. Breast Cancer Res Treat. (2023) 199:355–61., PMID: 36976395 10.1007/s10549-023-06923-1 PMC12529652

[2] Thomas HR Hu B Boyraz B Johnson A Bossuyt VI Spring L. Metaplastic breast cancer: a review. Crit Rev Oncol Hematol. (2023) 182:103924. doi: 10.1016/j.critrevonc.2023.103924, PMID: 36696934

[3] El Zein D Hughes M Kumar S Peng X Oyasiji T Jabbour H. Metaplastic carcinoma of the breast is more aggressive than triple-negative breast cancer: a study from a single institution and review of literature. Clin Breast Cancer. (2017) 17:382–91. doi: 10.1016/j.clbc.2017.04.009, PMID: 28529029 PMC5537027

[4] Basho RK Yam C Gilcrease M Murthy RK Helgason T Karp DD. Comparative effectiveness of an mTOR-Based systemic therapy regimen in advanced, metaplastic and nonmetaplastic triple-negative breast cancer. Oncologist. (2018) 23:1300–13 09. doi: 10.1634/theoncologist.2017-0498, PMID: 30139837 PMC6291334

[5] Wu Y Chen Z Li W Wang F Zhang Y. Primary squamous cell carcinoma of the breast: a case report and review of the literature. Front Oncol. (2023) 12:1033084. doi: 10.3389/fonc.2022.1033084, PMID: 36698422 PMC9869869

[6] Hu J Lang R Zhao W Jia Y Tong Z Shi Y. The mixed subtype has a worse prognosis than other histological subtypes: a retrospective analysis of 217 patients with metaplastic breast cancer. Breast Cancer Res Treat. (2023) 200:23–36. doi: 10.1007/s10549-023-06945-9, PMID: 37160814 PMC10224839

[7] Kowalewski A Klijanienko J. Cytologic analysis of metaplastic breast carcinoma: review of 66 cases diagnosed at the Institut Curie. Am J Clin Pathol. (2024) 161:4 30–435. doi: 10.1093/ajcp/aqad144, PMID: 37987613

[8] Cserni G. Histological type and typing of breast carcinomas and the WHO classification changes over time. Pathologica. (2020) 112:25–41. doi: 10.32074/1591-951X-1-20, PMID: 32202537 PMC8138497

[9] Lee JH Ryu JM Lee SK Chae BJ Lee JE Kim SW. Clinical characteristics and prognosis of metaplastic breast cancer compared with invasive ductal carcinoma: a propensity- matched analysis. Cancers (Basel). (2023) 15:1556. doi: 10.3390/cancers15051556, PMID: 36900347 PMC10000576

[10] Khoury T. Metaplastic breast carcinoma revisited; subtypes determine outcomes: comprehensive pathologic, clinical, and molecular review. Clin Lab Med. (2023) 43:221–43. doi: 10.1016/j.cll.2023.03.002, PMID: 37169444

[11] Jakubowska K Kańczuga-Koda L Kisielewski W Koda M Famulski W. Squamous cell carcinoma of the breast as a clinical diagnostic challenge. Mol Clin Oncol. (2018) 8:587–91. doi: 10.3892/mco.2018.1581, PMID: 29556390 PMC5844120

[12] Özkurt E Emiroğlu S Cabioğlu N Karanlık H Önder S Tükenmez M. Metaplastic breast cancer: mesenchymal subtype has worse survival outcomes. Breast Care (Basel). (2022) 17:554–60. doi: 10.1159/000525324, PMID: 36590148 PMC9801396

[13] Wang M He X Chang Y Sun G Thabane L. A sensitivity and specificity comparison of fine needle aspiration cytology and core needle biopsy in evaluation of suspicious breast lesions: a systematic review and meta-analysis. Breast. (2017) 31:157–66. doi: 10.1016/j.breast.2016.11.009, PMID: 27866091

[14] Ni Y Tse GM. Spindle cell lesions of the breast: a diagnostic algorithm. Arch Pathol Lab Med. (2023) 147:30–7. doi: 10.5858/arpa.2022-0048-RA, PMID: 35976671

[15] Djomehri SI Gonzalez ME Leprevost FDV Tekula SR Chang HY White MJ. Quantitative proteomic landscape of metaplastic breast carcinoma pathological subtypes and their relationship to triple-negative tumors. Nat Commun. (2020) 11:1723. doi: 10.1038/s41467-020-15283-z, PMID: 32265444 PMC7138853

[16] Abada E Kim S Dozier K Fehmi O Jang H Fehmi Z. Estrogen receptor status has no prognostic relevance in metaplastic breast carcinoma. Cancer Treat Res Commun. (2022) 33:100630. doi: 10.1016/j.ctarc.2022.100630, PMID: 36058202 PMC9742347

[17] Hennessy BT Giordano SH Broglio K Duan Z Trent J Buchholz TA. Biphasic metaplastic sarcomatoid carcinoma of the breast. Ann Oncol. (2006) 17:60 5–613. doi: 10.1093/annonc/mdl006, PMID: 16469754

[18] Han M Salamat A Zhu L Zhang H Clark BZ Dabbs DJ. Metaplastic breast carcinoma: a clinical-pathologic study of 97 cases with subset analysis of response to neoadjuvant chemotherapy. Mod Pathol. (2019) 32:807–16. doi: 10.1038/s41379-019-0208-x, PMID: 30723293

[19] Sherwani M Vohra L Ali D Soomro R Adnan S Idrees R. Clinicopathological features and survival outcomes of metaplastic breast carcinoma - an observational multi-centric study. Breast Cancer (Dove Med Press). (2023) 15:237–50. doi: 10.2147/BCTT.S398932, PMID: 37006839 PMC10065023

[20] Takala S Heikkilä P Nevanlinna H Blomqvist C Mattson J. Metaplastic carcinoma of the breast: prognosis and response to systemic treatment in metastatic disease. Breast J. (2019) 25:418–24. doi: 10.1111/tbj.13234, PMID: 30925636

[21] Rakha EA Tan PH Varga Z Tse GM Shaaban AM Climent F. Prognostic factors in metaplastic carcinoma of the breast: a multi-institutional study. Br J Cancer. (2015) 112:283–9. doi: 10.1038/bjc.2014.592, PMID: 25422911 PMC4453452

[22] Tower A Hughes J Moore L Srivastava K. Mixed metaplastic carcinoma of the breast: a case report. J Surg Case Rep. (2023) 2023:rjad144. doi: 10.1093/jscr/rjad144, PMID: 36926632 PMC10014167

[23] Pandey A Joshi K Moussouris H Joseph G. Case reports on metaplastic squamous cell carcinoma of the breast and treatment dilemma. Case Rep Oncol Med. (2019) 18:4307281. doi: 10.1155/2019/4307281, PMID: 31641544 PMC6766669

[24] Huang C Tian H Xu J Tong F Fang D. Metaplastic breast carcinoma with osseous differentiation: a report of a rare case and literature review. Open Life Sci. (2023) 18:20220640. doi: 10.1515/biol-2022-0640, PMID: 37528884 PMC10389674

[25] Chen IC Lin CH Huang CS Lien HC Hsu C Kuo WH. Lack of efficacy to systemic chemotherapy for treatment of metaplastic carcinoma of the breast in the modern era. Breast Cancer Res Treat. (2011) 130:345–51. doi: 10.1007/s10549-011-1686-9, PMID: 21792625

[26] Takuwa H Ueno T Ishiguro H Mikami Y Kanao S Takada M. A case of metaplastic breast cancer that showed a good response to platinum-based preoperative chemotherapy. Breast Cancer. (2014) 21:504–7. doi: 10.1007/s12282-011-0269-2, PMID: 21526425

[27] Alan O Telli TA Ercelep O Hasanov R Simsek ET Mutis A. A case of primary squamous cell carcinoma of the breast with pathologic complete response after neoadjuvant chemotherapy. Curr Probl Cancer. (2019) 43:308–11. doi: 10.1016/j.currproblcancer.2018.04.003, PMID: 29880396

[28] Vranic S Stafford P Palazzo J Skenderi F Swensen J Xiu J. Molecular profiling of the metaplastic spindle cell carcinoma of the breast reveals potentially targetable biomarkers. Clin Breast Cancer. (2020) 20:326–331.e1. doi: 10.1016/j.clbc.2020.02.008, PMID: 32197944

[29] Noro A Ishitobi M Hanamura N Kashikura Y Yamashita M Kozuka Y. A case of metaplastic squamous cell carcinoma of the breast that showed a pathological complete response after neoadjuvant chemotherapy with weekly paclitaxel. Am J Case Rep. (2022) 23:e935035. doi: 10.12659/AJCR.935035, PMID: 35017459 PMC8765086

[30] Nahhat F Doyya M Zabad K Ksiri H. Metaplastic breast cancer with a unique presentation and complete response to chemotherapy: a case report. BMC Womens Health. (2024) 24:285. doi: 10.1186/s12905-024-03134-8, PMID: 38734591 PMC11088025

[31] Ullah A Khan J Yasinzai AQK Tracy K Nguyen T Tareen B. Metaplastic breast carcinoma in US population: racial disparities, survival benefit of adjuvant chemoradiation and future personalized treatment with genomic landscape. Cancers (Basel). (2023) 15:2954. doi: 10.3390/cancers15112954, PMID: 37296916 PMC10251813

[32] Acar T Acar N Sezgin G Gokova MB Kucukzeybek BB Haciyanli M. What should be treatment approach in metaplastic breast cancer? a report of 5 cases. North Clin Istanb. (2018) 5:365–9. doi: 10.14744/nci.2018.09124, PMID: 30859170 PMC6372000

[33] Ysamaguchi R Horii R Maeda I Suga S Makita M Iwase T. Clinicopathologic study of 53 metaplastic breast carcinomas: their elements and prognostic implications. Hum Pathol. (2010) 41:679–85., PMID: 20153509 10.1016/j.humpath.2009.10.009

[34] Bourdeanu L Somlo G. Metaplastic breast carcinoma in the lungs: a case report. World J Oncol. (2013) 4:252–4. doi: 10.4021/wjon637w, PMID: 29147366 PMC5649851

[35] He X Ji J Dong R Liu H Dai X Wang C. Prognosis in different subtypes of metaplastic breast cancer: a population-based analysis. Breast Cancer Res Treat. (2019) 173:329–41. doi: 10.1007/s10549-018-5005-6, PMID: 30341462

[36] Lien HC Hsu CL Lu YS Chen TW Chen IC Li YC. Transcriptomic alterations underlying metaplasia into specific metaplastic components in metaplastic breast carcinoma. Breast Cancer Res. (2023) 25:11. doi: 10.1186/s13058-023-01608-5, PMID: 36707876 PMC9883935

[37] Polamraju P Haque W Cao K Verma V Schwartz M Klimberg VS. Comparison of outcomes between metaplastic and triplenegative breast cancer patients. Breast. (2020) 49:8–16. doi: 10.1016/j.breast.2019.10.003, PMID: 31675684 PMC7375639

[38] Voutilainen S Heikkilä P Bartkova J Nevanlinna H Blomqvist C Bartek J. Markers associated with genomic instability, immunogenicity and immune therapy responsiveness in metaplastic carcinoma of the breast: expression of γH2AX, pRPA2, P53, PD-L1 and tumor infiltrating lymphocytes in 76 cases. BMC Cancer. (2022) 22:1298. doi: 10.1186/s12885-022-10408-7, PMID: 36503417 PMC9743555

[39] Ladwa A Elghawy O Schroen A Abernathy K Schlefman J Dillon P. Complete response of triple-negative metaplastic carcinoma of the breast using pembrolizumab. Case Rep Oncol. (2023) 16:1129–1135. doi: 10.1159/000534146, PMID: 37900847 PMC10601781

[40] Adams S Othus M Patel SP Miller KD Chugh R Schuetze SM. A multicenter phase II trial of ipilimumab and nivolumab in unresectable or metastatic metaplastic breast cancer: cohort 36 of dual anti-CTLA-4 and anti-PD-1 blockade in rare tumors (DART, SWOG S1609). Clin Cancer Res. (2022) 28:271–8. doi: 10.1158/1078-0432.CCR-21-2182, PMID: 34716198 PMC8776596

[41] Carlucci M Iacobellis M Colonna F Marseglia M Gambarotti M Giardina C. Metaplastic carcinoma of the breast with dominant squamous and sebaceous differentiation in the primary tumor and osteochondroid metaplasia in a distant metastasis: report of a case with review of sebaceous differentiation in breast tumors. Int J Surg Pathol. (2012) 20:284–96. doi: 10.1177/1066896911417711, PMID: 21865268

[42] Coussy F El Botty R Lavigne M Gu C Fuhrmann L Briaux A. Combination of PI3K and MEK inhibitors yields durable remission in PDX models of PIK3CA-mutated metaplastic breast cancers. J Hematol Oncol. (2020) 13:13. doi: 10.1186/s13045-020-0846-y, PMID: 32087759 PMC7036180

[43] Shaker N Shafi S Parkinson B Chen W Parwani AV Ding Q. Wnt family member 9b (Wnt9b) is a sensitive and specific marker for triple-negative breast carcinoma including metaplastic carcinoma. Am J Surg Pathol. (2023) 47:47–54. doi: 10.1097/PAS.0000000000002001, PMID: 36525542

